# Overview of Materials Used for the Basic Elements of Hydraulic Actuators and Sealing Systems and Their Surfaces Modification Methods

**DOI:** 10.3390/ma14061422

**Published:** 2021-03-15

**Authors:** Justyna Skowrońska, Andrzej Kosucki, Łukasz Stawiński

**Affiliations:** Institute of Machine Tools and Production Engineering, Lodz University of Technology, ul. Stefanowskiego 1/15, 90-924 Lodz, Poland; andrzej.kosucki@p.lodz.pl (A.K.); lukasz.stawinski@p.lodz.pl (Ł.S.)

**Keywords:** structural materials, power hydraulics, hydraulic actuators, sealing system, coatings, surface modifications

## Abstract

The article is an overview of various materials used in power hydraulics for basic hydraulic actuators components such as cylinders, cylinder caps, pistons, piston rods, glands, and sealing systems. The aim of this review is to systematize the state of the art in the field of materials and surface modification methods used in the production of actuators. The paper discusses the requirements for the elements of actuators and analyzes the existing literature in terms of appearing failures and damages. The most frequently applied materials used in power hydraulics are described, and various surface modifications of the discussed elements, which are aimed at improving the operating parameters of actuators, are presented. The most frequently used materials for actuators elements are iron alloys. However, due to rising ecological requirements, there is a tendency to looking for modern replacements to obtain the same or even better mechanical or tribological parameters. Sealing systems are manufactured mainly from thermoplastic or elastomeric polymers, which are characterized by low friction and ensure the best possible interaction of seals with the cooperating element. In the field of surface modification, among others, the issue of chromium plating of piston rods has been discussed, which, due, to the toxicity of hexavalent chromium, should be replaced by other methods of improving surface properties.

## 1. Introduction

Hydraulic actuators are elements converting the energy of the working fluid into mechanical energy related to the reciprocating motion. The pressure of the working fluid acts on the piston and creates a force causing the piston assembly to move. As a result, the piston rod can perform useful work [1,2]. Hydraulic actuators are an executive element in power hydraulic systems. These structures have several advantages, which include the possibility of obtaining large working forces and low operating speeds [3].

The most general division of the actuators includes single-acting, double-acting, and rotary actuators [4]. A characteristic feature of single-acting actuators is the presence of one working chamber and the possibility of executive (active) movement only in one direction. The return movement can be carried out by an external force or a spring force. Double-acting actuators, in turn, are characterized by a working stroke in both directions.

**Figure 1:**
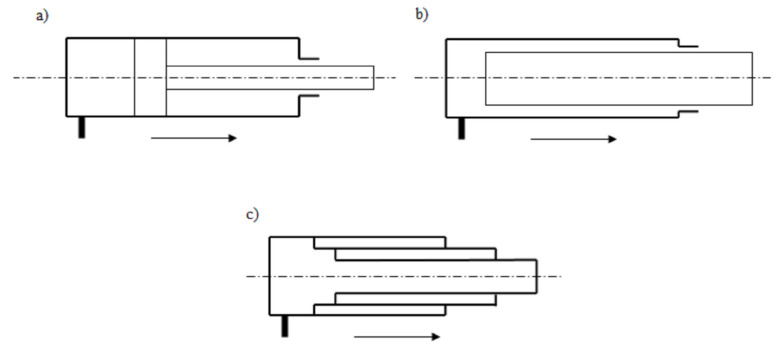
Types of single-acting actuators: (**a**) piston, (**b**) plunger, (**c**) telescopic.

Single-acting actuators are divided into (Figure 1):

- piston actuator;
- plunger actuator;
- telescopic actuator.

**Figure 2:**
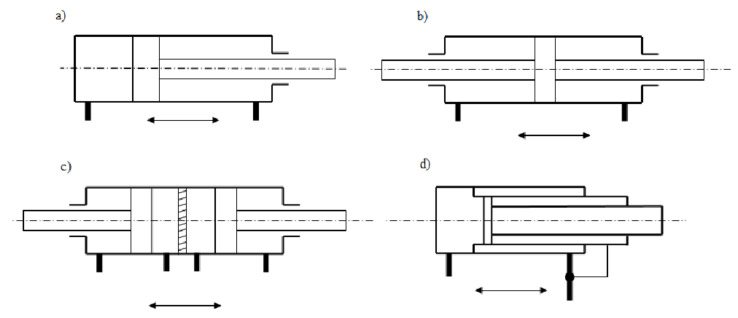
Types of double-acting actuators: (**a**) with one-sided piston rod, (**b**) two-sided piston rod, (**c**) multi-piston (double piston), (**d**) telescopic.

Double acting actuators are divided into (Figure 2):

- piston actuator with one piston rod (with one-sided piston rod);
- piston actuator with two-piston rods (with double-sided piston rod);
- multi-piston actuator;
- telescopic actuator.

Piston actuators are the most common type of actuators. They are characterized by the fact that at the end of the piston rod on which the piston is mounted.

In a plunger actuator, there is the so-called plunger instead of the piston assembly. A telescopic actuator consists of a set of several pipes, extending one from the other—such an actuator allows obtaining large strokes.

**Figure 3:**
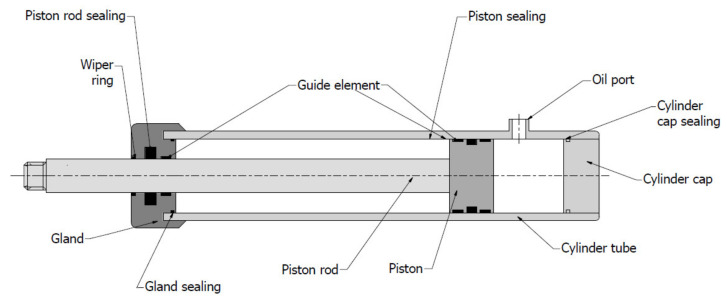
Design of hydraulic piston actuator.

The construction of a hydraulic actuator is described on the example of the simplest piston actuator (Figure 3).

The casing of the actuator units is a cylinder tube. Inside the tube, there is a piston rod with a piston fixed at the end. On one side, the cylinder is closed by the end cap, and on the other side, there is a so-called actuator gland [5,6].

The cylinder cap can be assembled with a pipe using a welded connection—this is the most common way. Another method is to use a threaded cap. In the case of the latter solution, it is necessary to use a static seal to ensure adequate tightness here [4,5].

The piston rod is the component that transfers the load to the receiving element. The gland is usually attached to the cylinder pipe using a threaded connection, which is why sealing between the two components is also necessary here. The gland contains the entire sealing system for the piston rod. Most often there are one or two seals to prevent the working fluid from flowing outside and a wiper ring to prevent contamination from being transferred from outside to inside the cylinder. Such a sealing system contributes to increase the life of the cylinder [5,7,8,9].

There are also guide elements of the piston rod in the gland. They must be sufficiently lubricated with the working fluid of the hydraulic system. These elements absorb radial loads and hold the piston rod axially [10].

The piston is usually connected to the piston rod using a thread connection. In this case, the sealing element must also be located here. Guide elements mounted on the piston transmit side forces and ensure that the piston is centred in the cylinder [5,10]. In addition to the guides, there are also sealing rings that prevent the working fluid from moving between the chambers. The piston seal acts as a pressure barrier which, for example, keeps the piston rod in a specific position [11]. Piston seals can be single- or double-acting. The thickness of the acceptable lubricant film depends on the design of the actuator. Single-acting actuators require a small oil film; in double-acting actuators, the oil film can be slightly thicker [10].

Hydraulic actuators are used in many branches of industry, often requiring large working forces. They are used in construction and transportation equipment (excavators, forklifts, telescopic handlers, basket elevators, booms) [2,12,13]. Industries, where hydraulic cylinders are used, include mining, robotics, and aviation [6,14]. Therefore, manufacturers of actuators are outdoing each other in new material solutions and methods of improving the operational parameters of actuators.

To systematize the materials and surface modification methods used for hydraulic actuators elements, an extensive review of the existing literature has been made, which allowed for the preparation of a work presenting the current state of art in the field of hydraulic actuators manufacturing. This paper focuses on several of the most important elements of hydraulic actuators: pistons, piston rods, cylinders, glands, bottoms, and seals. The most common causes of failure of these parts are described, with particular emphasis on operational factors such as abrasive wear and material fatigue. Errors during the component manufacturing stage were also noted.

In the next part, the analysis of materials used for selected parts of hydraulic cylinders was performed. Material groups such as iron alloys, light metal alloys, composites, and polymers are discussed. For each of the discussed materials, the most important mechanical properties are presented, such as tensile strength, yield strength, hardness, impact energy, or elongation at break.

In this review article, a division of materials and methods has been applied according to their purpose (for cylinders, pistons, etc.) Such systematics can be helpful when there is a need to know the latest manufacturing technologies applied to a given hydraulic actuators’ components. For each actuator part, materials were selected into subgroups such as ferrous materials, light alloys, etc. During the review, items from recent years were selected to discuss the latest publications presenting modern materials and methods of surface modification. The database of available materials is constantly being expanded, so it is important that the knowledge presented is as up-to-date as possible.

## 2. Selection of the Appropriate Material and Its Influence on the Operation and Durability of the Hydraulic Actuator

Many factors have to be taken into account during designing a hydraulic actuator. The aim is not only that each element performs its functions as well as possible, but also that all elements work together in the best possible way. Both mechanical (e.g., strength) and operational (utility) factors must be considered.

Many articles have been written about research on failures of hydraulic cylinders. Thanks to a detailed analysis, conclusions can be drawn that will help to avoid failures in future projects and to ensure that the cylinders will work as long as possible without failures. Prevention of failures usually involves changes in the actuator design, both structural and material.

The selection of appropriate material should be carried out taking into account the shape of the designed part, both in terms of external dimensions and cell structure. The issue of choosing the appropriate material for pistons, piston rods, and cylinders has been briefly discussed in [15].

Generally speaking, the selected material and shape should be complementary with the method of producing the given object during the design [16]. Even the selection of the best material with a badly applied manufacturing method may not bring the expected final effect.

The working conditions of the part, the loads acting on it, and possible exposure to harmful substances should be considered. Financial outlays are an important issue. It is worth looking for cheaper solutions and improving the technology in such a way as to ensure minimization of costs while obtaining similar or even better mechanical and functional properties.

Nowadays, when selecting the material, great emphasis is also placed on ecology. Progressive pollution of the environment entails the necessity of actions limiting the so-called carbon footprint, defined most simply as the impact of a product or process on the climate [17]. Industrial production contributes to the emission of a huge amount of greenhouse gases into the atmosphere [18], so there is a need to look for the most environmentally friendly methods of manufacturing products.

Additionally, a tendency to reduce the weight of machine elements is observed. Less weight is usually associated with energy savings, increased efficiency, and reduced financial outlays [19,20,21,22]. Reducing the weight of an element by changing the material can be carried out, for example, by changing steel to light metals or plastics. However, the new material must meet the needed requirements for the designed part—it must not be too weak or, for example, not resistant to working conditions.

**Figure 4:**
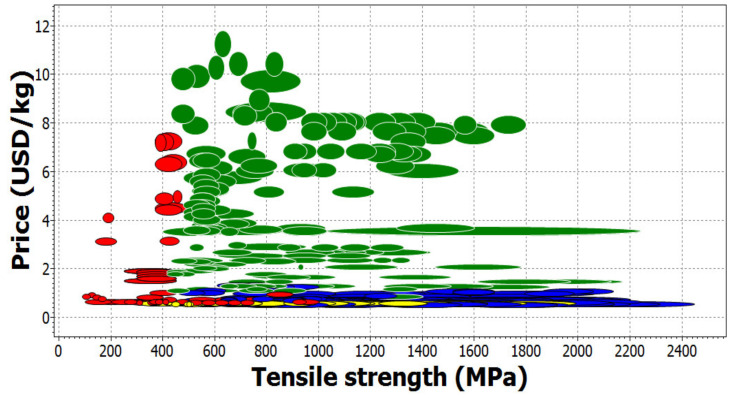
Example of a material chart obtained in the CES EduPack software.

There are many tools for selecting materials. The optimum material is one that has the right values for the relevant parameters for the specific application. An interesting tool is the CES EduPack software, which allows analyzing materials and drawing material diagrams taking into account the values of various temperature, mechanical, and economic parameters. An example of such a material chart can be found in Figure 4. In this case, it is a graphical representation of different iron alloys in terms of selected parameters: tensile strength (horizontal axis) and price (vertical axis). In this case, stainless steel is marked in green and cast iron in red. Blue is responsible for low alloy steels and yellow for carbon steels. Each dot or oval represents a different material.

**Figure 5:**
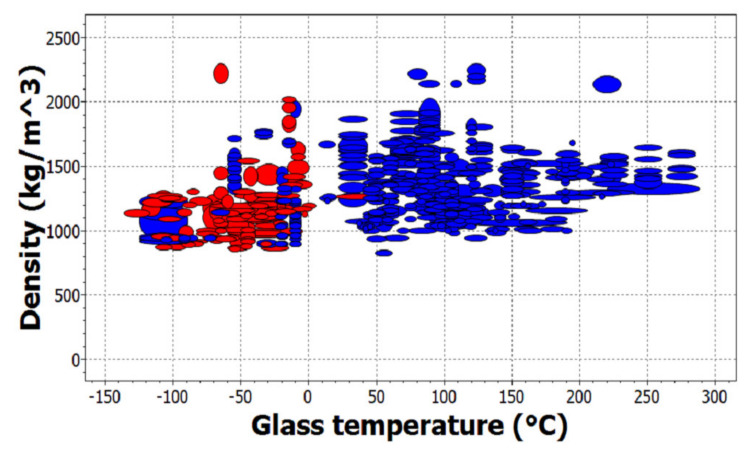
Example of a material chart obtained in the CES EduPack software.

In Figure 5, there is another graphical representation of materials. The chart shows polymers in terms of density and glass temperature. Elastomers are marked in red and thermoplastic polymers are marked in blue.

The material properties can be modified by appropriate heat or heat-chemical treatment. Another way is to apply coatings required to meet several requirements, such as reduced friction, vibration damping, appropriate hardness, abrasion resistance, and good adhesion to the substrate [23].

## 3. Failures of Hydraulic Actuators

Failures of hydraulic actuators may result from various reasons. These reasons are either related to the actuator manufacturing process or operation. The literature extensively describes research on these breakdowns, and solutions were often proposed that could prevent similar defects in the future.

The most common causes of damage and failure of hydraulic actuators are three factors: abrasive wear, material fatigue, and friction [24].

Abrasive wear can lead to a risk of internal leakage and a decrease in the volume efficiency of the actuator [25]. Material fatigue (associated with the occurrence of variable loads) contributes to so-called fatigue cracking, hence the need to design the actuators in such a way as to ensure the highest possible resistance to this type of cracking [26].

Failures caused by fatigue are also investigated in [2]. During the fatigue failure analysis, it is necessary to locate the places where the stress concentration in the actuator is high and may negatively affect the strength. Usually, such concentration occurs in places of geometric discontinuities [27].

It is also worth mentioning the issue of uniformity of working movements in the actuator. It is important because it affects the accuracy of piston rod positioning, the uniformity of the contact force, and the safety of the user of the hydraulic actuator. The uniformity of piston movement is related to the type of sealing and piston rod guiding elements, piston rod load, and wear of the actuator elements. Non-coaxial load influences the change in the uniformity of piston movement [14,28].

The work [29] discusses a case of damage to a hydraulic actuator caused by design errors of the hydraulic system and operator’s errors during manual control of the machine operation.

Another cause of problems with the actuator are vibrations and oscillatory movements. The article [30] proposes a solution in the form of vibration reduction using rotary viscous dampers.

Corrosion of cylinders, pistons, and piston rods can be a significant problem. As a result, it is necessary to apply corrosion-resistant materials on the actuator elements and/or to apply an appropriate anti-corrosion coating [31].

In the article [23], it has been noted that corrosion and tribological extortion entails the risk of unfavourable structural changes in the material used for the actuator, which leads to a deterioration in its functional properties and, consequently, to system failure.

### 3.1. Failures of Cylinders

Failures of hydraulic cylinders can result from many factors. Most often it is material stress caused by high pressure, loss of stability, corrosion, and fatigue cracking [3,12,26].

The fact of high pressures in a hydraulic system should be taken into account during the cylinder design process. Cylinders under the influence of pressure may suffer from deformation manifested by e.g., change of diameter [32]. A pipe exposed to high pressures may swell. The solution is to select appropriately thick walls, which will prevent deformation and thus reduce the risk of seal failure and loss of tightness. Welding joint failures also often occur in high-pressure cylinders. The material of pipes working in such conditions is required to have a high yield strength, good weldability, and right impact capabilities [33].

Cylinders can be subject to axial and radial forces and fretting vibrations. That results in abrasive wear on the pipe surface [34]. It can significantly accelerate corrosive wear.

Fatigue cracking can occur in the cylinders due to stress concentration at welding joints, e.g., at oil connections [26,35]. This leads to oil leakage near the working fluid ports. The article [35] proposes the introduction of a washer made of heat-resistant material or glue to fill the gap between the cylinder surface and the oil inlet. By protecting the gap against oil ingress, the propagation of fatigue cracks in the joint can be prevented.

### 3.2. Failures of Piston Rods and Pistons

Piston rods are those elements of hydraulic actuators which most often fail [36]. This is because these components are often exposed to an aggressive environment and compressive cyclic loads [37].

Because the piston rod slides out of the cylinder, it is highly exposed to external factors. Temperature changes, precipitation, dirt, and dust often cause damage to its surface, which leads to failures that can be dangerous for people working nearby [13].

The forces in the piston rod should be applied axially, but sometimes lateral forces may also occur, which is not beneficial for the piston rod operation. This can occur either intentionally or accidentally. The piston rod is subjected to quite high tensile or compressive stress. In the case of single-acting actuators, the piston rod is only subjected to compressive stress, so only buckling is considered in the strength calculation. This is different for double-acting cylinders. Here, there are both compressive and tensile stresses, so fatigue strength must also be taken into account in the strength calculations [1,38].

In an element as important as the piston rod, the correct choice of surface modification is crucial. The article [39] analyzes the piston rod failure related to its fracture. The results showed that the crack could have been the result of improperly selected heat treatment, which led, among others, to susceptibility to stress corrosion cracking (SCC) and low toughness of the piston rod material.

In [40], the piston rod failure related to the crack that appeared in the centre of the welding joint and then propagated further was described. The results showed that the carbides formed during the nitriding process contributed to the brittleness of the joint. This phenomenon resulted from the coexistence of two factors: the use of unsuitable welding material and subsequent nitriding of the piston rod.

The issue of cracking was also examined in works [37,41], where it was noticed that the segregation of copper and nickel in the material of the piston rod can cause cracking resulting from improperly performed heat treatment.

Another piston rod failure was described in the article [42]. Here, the destruction resulted from fatigue related to the stress concentration at the rounding point on the piston rod (so-called “fillet”). Fatigue life reduction was also featured in the article [43]. In this case, the stress concentration caused the change of the thread used to connect the piston rod with the piston to a smaller pitch thread. During operation, the piston rod is also exposed to abrasive wear [44].

The piston and piston rod together form a unit, so the materials used to make the pistons and piston rods should have similar hardness and high impact strength [33]. The piston is much less likely to fail than the piston rod, which is a result of different operating conditions. Scratches or pits appear most often on the piston surface and they disturb the degree of geometric accuracy and may contribute to the loss of tightness. In [45], it was noted that the critical point may be the piston edges.

### 3.3. Failures of End Caps and Glands

The end cap and the gland are not among the most loaded elements of the actuator, but there are a few things to consider during designing them.

If the cylinder cap is welded—a crack may occur at the joint. The situation of that failure is described in the article [2]. In this case, the welding joint is fatigued, mainly due to cyclical loads. On the other hand, in the case of the ends connected to the cylinder by means of a thread, the problem may be caused by leakage of working fluid. In such cases, it is necessary to use a static seal at this point.

The gland is usually fastened with a thread. In this case, also it is usually necessary to use a seal here to prevent working fluid from leaking from the actuator chamber. It is worth mentioning that in the case of higher pressures, screwing the gland is not recommended due to the risk of swelling of the cylinder pipe and the possibility of loosening of the threaded connection [33]. In such cases, e.g., structural modifications of the existing threaded connection are made.

The article [46] describes the failure of the hydraulic actuator gland in aviation related to leakage. It was shown that the pressure bolts broke during the machine start-up test. It was related to material fatigue. Measures are proposed here to increase the fatigue strength of these bolts, such as modifications to the way the bolts are manufactured.

### 3.4. Failures of Sealing Systems

Sealing systems are often a critical element of hydraulic cylinders [47]. The main mechanisms responsible for seal failures are swelling, thermal degradation, deformation, and wear associated with contact with the other surface [48]. Destruction of seals leads to loss of tightness and, as a result, to leakage of working fluid.

Seals are exposed to many negative external factors such as high temperature, radial loads, aggressive environment, and harmful substances. During designing the seals, it is necessary to take into account the pressure range of the fluid in the system and possible pressure peaks, the temperature range, the speed of the piston rod, the condition of the cooperating surface, and the type of working fluid. All these factors affect the durability of the sealing system and the performance of the entire hydraulic system [10,49,50].

The basic requirements for the dynamic sealing system in cylinders are of course low friction coefficients and leaks close to zero [51]. The issue of friction is related to the performance of seals and their durability [52]. Seals must be designed with materials of appropriate module and hardness [10]. The design of the seal should ensure appropriate resistance to friction and corrosion, easy assembly/disassembly and a possibility to work in a wide temperature range [6]. There should be a small layer of working fluid on the piston rod seal. The lubricating film reduces friction, which contributes to increasing the life of the seals. Another advantage is the prevention of corrosion on the piston rod surface [5,10,48,53].

In [54], it was noted that the friction force and sensitivity to load variation is related to the type and shape of the seal used. It was also found that friction may negatively impact the accuracy of position adjustment.

Apart from friction, the wear of piston rod seals often results from oil contamination, which is not without influence on the life of the entire system [55].

The piston seal separates two chambers of different pressure. If the seal expands too much under the influence of temperature and presses too hard against the inner surface of the pipe—the lubricant may be completely removed, causing the seal to wear. Therefore, the seals used for large diameter and high-pressure pistons should be reinforced with different materials. The use of fabric can reduce thermal expansion and compensate for the excessive pressure of the seal on the sliding surface of the hydraulic cylinder. Additionally, it is important that the piston seal should be symmetrical to the transverse axis of the piston, otherwise a loss of tightness could occur when the piston is loaded [32].

The article [50] examines the behaviour of seals on chromium plated piston rods. The tests showed that the seal material and the condition of the piston rod surface have a key influence on friction and wear of the seals. The issue of friction can only be neglected if the hydraulic power of the actuator is sufficiently high. For more demanding applications with the necessary high positioning and control accuracy, tribology cannot be neglected.

The article [56] confirms that the material used for the seals and the pressure conditions in the chambers influence the dynamic friction conditions.

The research described in [57,58] showed that tribological issues and tightness of the system are influenced by the roughness of cooperating surfaces.

The high roughness of the sliding surface causes a thick lubricating film. The edges of the seals wear out quickly as they come into contact with the roughness peaks. Too small roughness in turn makes the film thin, so the friction forces are much higher [5].

The article [59] discusses the influence of anisotropic surface roughness on friction at the contact between the two elements. Anisotropic roughness is created during the machining of a part. It was shown that the friction force decreases if the cylinder surface is grooved perpendicularly to the direction of motion.

The stick-slip phenomenon occurring at low sliding speeds is also important. It consists of causing vibrations by changing the friction force. In the article [60], it is noted that the phenomenon was related to the transfer of carbon monoxide from carbon steel to the sealing surfaces. This leads to noise and accelerates the wear of sealing elements.

Problems with seals such as extrusion and cracking often result from excessive pressure [33]. In the article [61], it is noted that high pressure in the actuator system used in aviation can cause the formation of thicker fluid layers and thus more effective lubrication.

It is noted that the design of the wiper ring has a major impact on the control of leaks from the actuator gland [9]. It seems obvious that double lip scraper rings can cause less leakage than single lip ones. Unfortunately, however, double lip wipers, due to their design, can cause the wiper ring or seal ring to be ejected from the groove.

Sealing failures can lead to dangerous failure of the entire hydraulic system, so it is important to regularly check the condition of the seal and replace worn seals [48].

## 4. Materials and Surface Modifications Used for Hydraulic Actuator Elements

### 4.1. Cylinders

#### 4.1.1. Materials Used for Cylinders

A variety of different materials are used for the cylinders, from metal materials to composites and polymers. Most often, the cylinders are made of steel or aluminum. At pressures lower than 10 MPa, cast iron cylinders are used. At pressures higher than 20 MPa, they are made of seamless steel pipe. For pressures above 20 MPa, the pipes are made of steel or forged steel [6].

**Figure 6:**
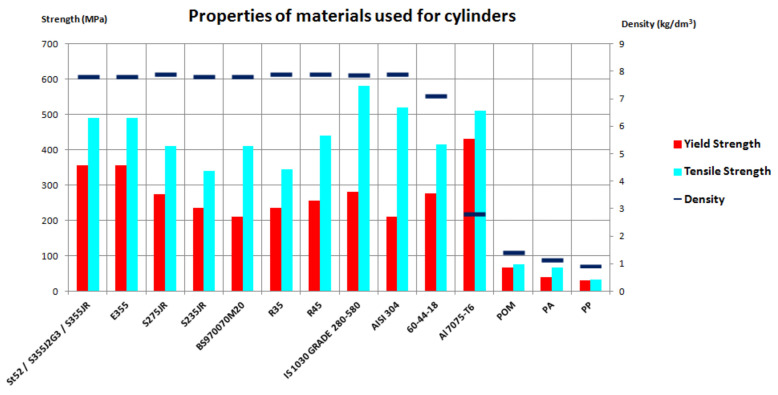
Chart of basic properties of the materials used for the cylinders.

The properties of the discussed metals and polymer materials are described in Table 1 and Table 2. The basic data is also presented graphically in Figure 6. The chart clearly shows the difference in the density of steel and polymer materials.

One of the most popular cylinder steels is steel St 52 [26,27,35]. It is steel belonging to the grade of low-carbon structural steel. It is characterized by good weldability, machinability, and does not require special treatment for both processes. This material in the context of this application is also described in the article [62]; however, there is a designation according to European standards: S355J2G3. Similar material is also described in [22], where it appears as S355JR. The latter two types of steel differ only permanently in the temperature of the impact test, as indicated by the mark at the letter “J.” Another equivalent of this steel, also used for cylinder tubes, is E355 [2]. It is quality steel, often used for tubular elements.

Cylinders are also made of S275 JR [20,63] and S235 JR [21]. Both have slightly lower yield strength than S355 (Table 1).

Cylinders made of another low-carbon steel are mentioned in [45]: BS970070M20. This material is also available under a different designation: C22E. This steel was selected for testing in this article because of its appropriate yield strength and tensile strength. Moreover, its advantage is low price. Other materials used for cylinders are unalloyed steels with a special purpose: R35 or R45 [64]. Cylinders can also be made of IS 1030 GRADE 280-580 [65] or AISI 304 stainless steel [21]. The latter, however, is used for special applications or those working in difficult conditions.

In the article [66], an analysis of a selection of suitable material for the cylinder pipe was carried out. After establishing requirements such as corrosion resistance, high strength, low density, good machinability, and low cost, the most optimal material was selected: spheroidal cast iron 60-40-18. It is a good choice in hydraulic systems with relatively low working pressures. Moreover, the use of cast iron allows avoiding noise caused by excessive vibrations, as this material, like any cast iron, has the ability to dampen them [67].

Apart from ferrous materials, the tendency to reduce mass determines the use of light metal alloys, e.g., aluminum, for cylinders. In [68], AlZnMgCu cylinders are mentioned—this is a group of aluminium alloys, in which zinc is the dominant additive. To this group belongs the material Al 7075-T6, which was described in many articles.

In [21], the steel materials were compared with Al 7075-T6 and a composite material consisting of 40% epoxy resin and 60% carbon fibers. Comparing the cylinder made of structural steel and this composite material, the mass was reduced by as much as 96% while maintaining appropriate mechanical properties. Unfortunately, the price of the composite cylinder is even several times higher than for the other materials tested. A good solution seems to be the use of aluminum, which reduces the weight of the cylinder by 91% compared to steel, and in economic terms, it more than doubles the price of the cylinder.

In [22], it was noted that the change of material from structural steel to Al 7075-T6 resulted in a significant reduction of the cylinder weight, which had a positive impact on the degree of the ecology of the hydraulic system as well as on its power demand. In [63], similar tests were carried out, but here the Al 7075-T6 aluminium pipe was wrapped in a composite consisting of epoxy resin and carbon fibers with the same proportions as in [21]. Such a solution was chosen to avoid damage caused by seals slipping on the inner surface of the pipe. A similar construction was also discussed in works [19,20].

In [69], the pipe is made from a thin S355 steel insert on the inside and is wrapped with CFRP (Carbon Fiber Reinforced Polymer) on the outside. The use of the insert was to provide the sealing system with appropriate tribological working conditions. This approach reduced the weight of the cylinder by several dozen percent. CFRP material has high strength, is resistant to corrosion and fatigue. Additionally, it has a low density. As the name suggests, it consists of carbon fibers embedded in a polymer resin, in which the carbon fibers act as a strengthening material and the polymer resin acts as a matrix holding the fibers [70]. The article [71,72] also discusses the application of CFRP to hydraulic cylinders. The paper [71] points out that the application of the composite for the cylinder pipe will allow for significantly reducing the weight; however, it will bring additional tribological problems in the piston-cylinder contact, hence the need to find a suitable method of manufacturing such cylinders. In the article [72], it was noted that in some situations steel inserts in a polymer tube may be damaged due to critical stress caused by the difference in mechanical properties of the metal and CFRP. The article notes the necessity of stiffening the structure. The application of nanostructured epoxy resin gelcoat made of Al2O3 nanoparticles inside a multilayer CFRP cylinder was proposed as a method. The inner surface of such a cylinder proved to be very durable.

The article [73,74] present prototypes of cylinders made of POM (polyoxymethylene) plastic. Such an approach was explained by many advantages of this type of materials: reduction of weight, an increase of vibration damping, and thus reduction of noise and nuisance to the environment. POM plastic shows adequate mechanical strength and heat resistance. Plastics can provide better tribological properties and resistance to aggressive environments.

In [75], the use of polyamide (PA) and polypropylene (PP), of which prototype hydraulic cylinders are made, is described. PA is a polymer with good mechanical properties and abrasion resistance, but can, unfortunately, absorb water. In turn, PP is a material characterized by chemical inertia and very good fatigue strength [76].

#### 4.1.2. Surface Modifications of Cylinder

The inner surfaces of the cylinders are ground and honed to achieve a smooth surface. However, sometimes machining proves to be insufficient. The performance of most steels used for hydraulic cylinders is improved by additional surface modification.

In [64], the technology of production of hydraulic cylinders is described, which is a combination of machining and forming (burnishing). Burnishing can be carried out with simultaneous rolling of the sleeve or after the manufacturing process. It enables to skip the honing stage. Burnishing allows obtaining high smoothness of the surface layer and additionally strengthens it. The surface becomes more resistant to abrasive wear and fatigue. The pressing process is also economical as it can be carried out simultaneously with other processes.

The properties of the steel used for cylinders, such as hardness, can also be improved by carburizing [45]. After the carburizing process, hardening is carried out, which directly leads to increased resistance. The surface layer of the steel becomes wear-resistant and retains its mechanical properties at the same time [77].

For special applications, the inner surface of the cylinder can be chrome plated—this is the case with telescopic cylinders, for example. Such a coating makes it possible to give it abrasion resistance. Its other properties and the reasons for the gradual abandonment of this technique are described in more detail in Section 4.3.2.

In [24], the application of n-Al_2_O_3_/Ni–Co nanocomposite coatings on hydraulic cylinders by brush plating was mentioned to increase their resistance to wear.

Hydraulic cylinders can also be coated with zinc or tin coatings on the outside. The main role of such coatings is to give protection against corrosion. This is especially important for cylinders working outside and exposed to adverse weather conditions.

In [78], the application of DLC (Diamond-Like Carbon) coatings on hydraulic actuator tubes is also mentioned. These coatings are resistant to harmful substances, characterized by low friction coefficient and high hardness as well as resistance to wear and corrosion.

### 4.2. Pistons

The pistons are usually made of steel or cast iron [4]. It must be a high-quality material to allow for the longest possible trouble-free operation.

In [79], spheroidal cast iron 65-45-12 for piston application is mentioned. It is a good alternative to carbon steel. The graphite content facilitates processing. Furthermore, ductile iron is lighter than steel.

The pistons of hydraulic cylinders can be made of C35 or C45 steel [6]. These are unalloyed steels that are very easy to heat treat, but difficult to weld. Other steels are S275 JR [63], S 355 JR [22], and BS970070M20 [45]. These are structural steels already mentioned in the hydraulic cylinders. Stainless steels are used for pistons (e.g., in special purpose cylinders) [80].

Apart from ferrous materials, light metals such as Al 7075-T6 [63,69] are also used in the context of this application.

Currently, research is being conducted on the application of composite and polymer materials in pistons. The article [73] proposes a piston from POM.

Pistons are rarely subject to surface modifications. Sometimes it is possible to improve their surface parameters by applying DLC coatings [78].

**Figure 7:**
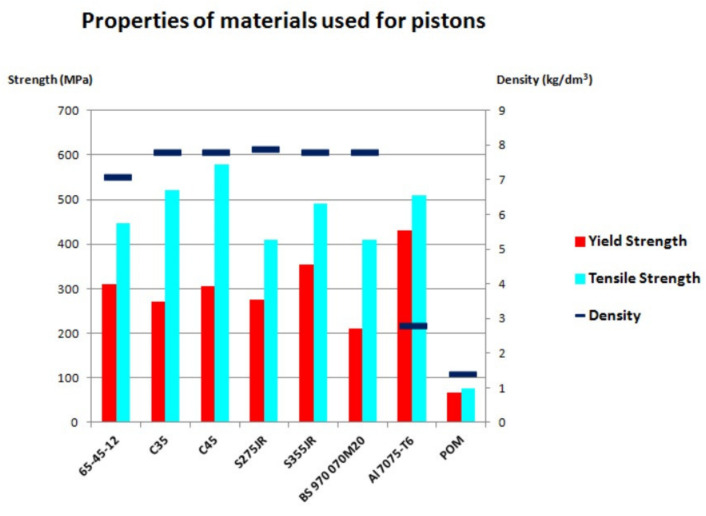
Chart of properties of the materials used for the pistons.

The properties of the discussed materials are described in Table 3 and Table 4. The basic data is also presented graphically in Figure 7.

### 4.3. Piston Rods

#### 4.3.1. Materials Used for Piston Rods

The piston rod, as a quite demanding element, must be made of good quality material with appropriate parameters. Both unalloyed and alloyed and even stainless steel are used for the piston rod.

In [21], S235 JR steel is used for the piston rod. In [63], steel with a slightly higher yield point was selected: S275 JR. In [81], the use of other structural steel was described: S355J0. Its other designation is 18G2A.

Many papers discussed piston rods made of steel C45 [43,44,82,83]. In [6], apart from the mentioned steel, C35 is also used, and if the influence of vibrations is high, even C55 or 40Cr. In [84], piston rods made of the equivalent steel 40Cr, 40X are used. In [1,38], C45E is used.

The steel commonly used for the piston rod can also be 40HM (42CrMo4) [42,69,81]. Other materials are 20MnV6 [85], 19MnVS6, 38MnVS6 [1], or 30CrNiMo8 [70]. BS970070M20 [45] and similar BS970070M55 (other designation: C55E) [40] are also applied.

For more demanding applications, stainless steel is used. These are 17-4PH [41], AISI 304 [21], and AISI 410 [39]. The latter belongs to the group of martensitic steels, which are mechanically durable and at the same time resistant to corrosion. Their resistance depends strictly on chromium and carbon content and processing conditions. Unfortunately, these materials belong to the group of soft steels [86].

Apart from steel, piston rods can be made of aluminium alloys [68]. In [20,21,63], the piston rod is made of Al 7075-T6. In [73,74], the piston rod is made of POM. Another material, still in the testing phase, is a composite of epoxy resin and carbon fibers [21].

**Figure 8:**
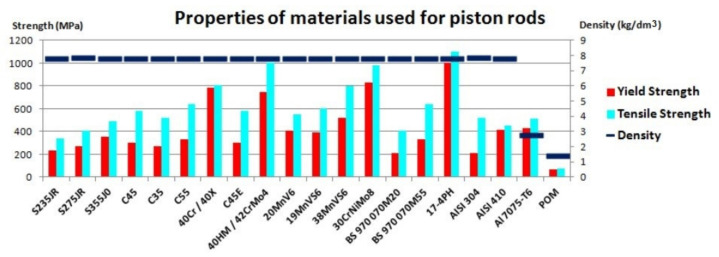
Chart of properties of the materials used for the piston rods.

The properties of the discussed materials are described in Table 5 and Table 6. The basic data is also presented graphically in Figure 8.

#### 4.3.2. Surface Modifications of Piston Rods

The surface of the piston rod is modified very often by heat- and heat-chemical treatment or by coating.

In [40,87], quenching and tempering was proposed as a treatment for the piston rod. Improvement is a process of combining hardening and high tempering. Its aim is to achieve high impact strength and ductility with appropriate hardness and strength values [88].

In [89], there is talk about induction hardening of the hydraulic cylinder piston rod. This method allows increasing surface resistance to damage. In [41], the precipitation hardening was proposed to improve the surface parameters of the piston rods and obtain high strength of the layer.

Among the coatings, a definitely basic modification is hard chromium plating [4,9,38,50,84,90]. Such a coating is applied electrolytically in a bath [91]. The action of direct current causes the deposition of metal ions of the coating on the steel surface.

Chromium plating allows obtaining a coating resistant to wear and corrosion, even in aggressive environments. The hard chrome layers are characterized by high hardness and low friction. However, the accuracy of application is important, as there is a risk of micro-cracks weakening the properties of coatings [92]. The solution may be to apply a layer of nickel under the chromium layer, which additionally improves the corrosion resistance [38].

Unfortunately, chromium coatings have their disadvantages. Already during the coating process, there is a risk of chrome poisoning by the process personnel. This metal is toxic and may cause respiratory damage, asthma, or cancer [93]. The process absorbs large amounts of energy and generates dangerous waste, which is difficult to utilize [94]. In many countries, it is recommended to abandon hexavalent chromium coatings by standards introducing limits for this element or completely banning its use in some applications [95,96,97]. Hence, there is a need to find alternatives to this method of surface modification.

The issue of alternative surface modifications is described in detail in the article [95]. The main method is the use of one of the varieties of thermal spraying—HVOF (High Velocity Oxygen Fuel) process, which can give corrosion and wear resistance even better than galvanic chromium plating. This method is based on the fact that the particles of the applied material are raised in a gas stream with a speed several times higher than the speed of sound. Such high speed allows limiting unfavourable changes in the chemical composition of these particles. Accelerated particles affect the surface of the substrate and result in a coating [77,98]. This method can be used to obtain homogeneous coatings with low porosity, good adhesion to the substrate, and high strength. The process is more efficient than galvanic chromium plating and does not produce so much waste. However, the cost may be even twice as high as for electrolytic chromium plating [94,95,97,99].

Examples of coatings used on piston rods produced by HVOF and giving similar features as electrolytic chrome plating are: WC-10Co-4Cr, Cr_3_C_2_–NiCr, and WC/Co/Cr [95].

Another alternative is APS (Atmospheric Plasma Spraying). The process allows obtaining coatings of similar structure and porosity to HVOF. It consists of melting the material for the coating in electric arc plasma [77]. In the case of plasma spraying, it is possible to combine materials in one layer and control the parameters of the coating relatively precisely. Plasma spraying has the advantage over HVOF that it can also be used for internal surfaces, such as cylinders. Coatings that can be applied to the piston rod using the APS method are, e.g., Cr_2_O_3_–SiO_2_–TiO_2_ [95].

HVOF coating as an alternative to chromium plating is also given in [83]. The possibility of applying Fe/TiC coatings produced with HVOF to the piston rods was investigated, and its properties were compared with hard chromium coatings applied electrolytically and with WC/CoCr coatings applied with HVAF (High Velocity Air Fuel) process. Studies have shown that Fe/TiC coatings showed similar anti-wear and anti-corrosion properties as the reference coatings, but their application was much more economical. The method of thermal spraying for modification of the piston rod surface was also mentioned in [87].

In [100], it was noted that the application of protective layers melted from cored wires (CW) under the flux layer is more ecological and economical than galvanic chromium plating. The article examines different layers embedded in the mentioned technology, differing in phase structure. It was shown that with an increase in the percentage of ferrite in the structure, the corrosion resistance of the austenitic matrix increases. Among the investigated coatings, the most resistant to corrosion turned out to be the core wire coating with an only ferritic matrix structure.

In [50], the application of CVD (Chemical Vapour Deposition) and PVD (Physical Vapour Deposition) coatings, laser coating, and thermal spraying were indicated as an alternative to chromium plating. Surface treatment technologies are also proposed as a combination of gas nitrocarbonation, plasma nitrocarbonation, and oxidation. This provides a layer that protects against corrosion and wear and has tribological parameters. Nitrided piston rods were also mentioned in [81].

In [101], it was proposed to apply a nanostructured tungsten carbide coating deposited with CVD on metal elements in contact with seals in order to minimize possible leaks, among others in actuators. Such a coating allows obtaining high resistance to abrasion and corrosion and general protection of surface roughness parameters of the substrate. All this has a positive effect on the durability of seals. It has been noted that this coating can also be a very good alternative to hard chrome coatings.

In [24], as an alternative to chromium coatings on the piston rod, a n-Al_2_O_3_/Ni–Co nanocomposite coating produced in the process of brush plating was proposed. This method belongs to galvanic methods. It can be used to repair damages in chromium plated piston rods. Such a coating gives much higher wear resistance and is tribologically similar to hard chromium coatings.

In [44], the method of repairing piston rods that have been corroded by powder welding (Plasma-Powder Surfacing) with a Fe–C–Cr–V coating is described. Conventional arc welding (AS, Arc Surfacing) could not be applied due to the risk of deformation of a relatively small diameter bar. Corrosion-resistant material, chrome-vanadium cast iron 315Kh19F3 grade was used as a padding weld.

In [25], the application of DLC coatings for hydraulic actuator components, which are covered by the concept of sliding motion, was examined. An example of such an element is the piston rod. In [78], the application of DLC coatings for the piston rod of a hydraulic cylinder used in construction machines was mentioned. It was related to very good parameters of these coatings in terms of resistance to wear and aggressive environment [23].

In [102], the DLC coating was compared with TiN coating. Both are characterized by high mechanical strength. TiN has better wear resistance, but DLC has better tribology and corrosion resistance.

### 4.4. End Caps and Glands

Glands and end caps of hydraulic actuators are described and tested relatively rarely. Both of these elements are indirectly responsible for the tightness of the actuator, so great care must also be taken to manufacture them.

The caps are often made of the same material as the cylinder. This can be low carbon steel S355 [69], S355 JR [2], or S275 JR [63]. In [20,63], Al 7075-T6 alloy is additionally proposed for this element.

In the work [65], IS 1030 GRADE 280-580 was used for the cylinder cap. In [45], BS970070M20 was proposed. The article [4] also mentions the cast iron caps. Quite a modern and still being tested material is POM [73,74]—such elements are used for cylinders made of plastic.

As far as the gland is concerned, it can be made of steel or aluminum. The gland made of S275 JR steel is described in [63]. In industry, these parts are also made of C45, 42CRMo4, AISI 304, or G25 cast iron.

Aluminum Al 7075-T6 for glands was used in [20,63,69]. In [73], the application of POM type plastic is mentioned.

**Figure 9:**
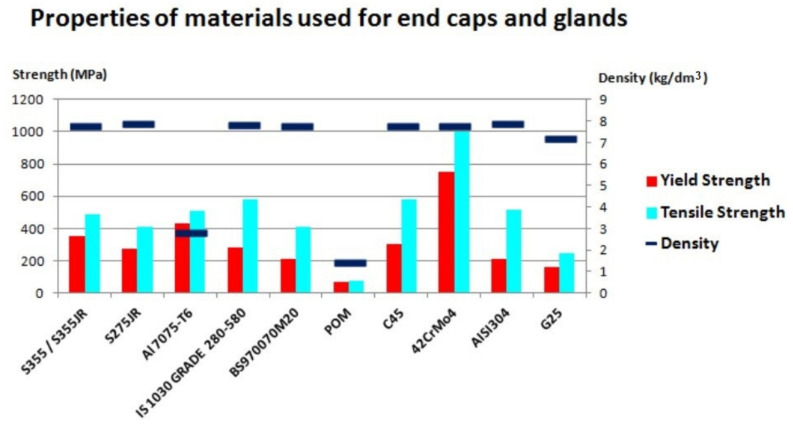
Chart of properties of the materials used for the end caps and glands.

When it comes to surface treatment, glands can sometimes be modified with chromium-containing coatings, which protect the elements against corrosion [95]. Table 7 and Table 8 contain properties of materials used for the end caps and glands. The basic data is also presented graphically in Figure 9.

### 4.5. Seals

The most general division of seals includes static and dynamic seals. Static seals occur mainly between the screwed-in bottom or gland and the cylinder pipe [11]. Additionally, the design solution may require the use of seals between the piston and the piston rod. When selecting a suitable sealing material, it must be taken into account whether the seal is only to protect against leaks or to work well with the moving surface and ensure the highest possible tightness in dynamic conditions [5].

As far as the shape of the seal is concerned, a lot depends on the operating conditions of the sealing elements. Seals operating under static conditions most often occur in round cross-section varieties (so-called O-rings). This is because a complex sealing profile is not required here [1]. Seals of this type may also act as pressure rings.

In [54], three types of piston sealing systems used were analyzed. These are:

1. combined sealing system consisting of:
  - flexible sealing element;
  - two rings to prevent squeezing of the seals;
  - two piston guide rings.
2. seal with a Glyd ring consisting of:
  - Glyd ring, pre-compressed by the O-ring;
  - backup ring to protect against contact between metal and metal.
3. U-profile sealing for double-acting actuators consisting of:
  - U-profile seals;
  - backup ring to protect against contact between metal and metal.

In the same work, a system of three seals was proposed for the piston rod:

- wiper ring;
- a standard U-profile piston rod seal;
- guide ring.

Materials used in power hydraulics are required to be insensitive to greases or oils and have good resistance to abrasion, wear, and high pressure. At the same time, a small coefficient of surface friction of such elements is important [10].

Among the materials used for sealing systems, most come from the polymer category. These are different types of rubber. The simplest polymer division divides these materials into [76,103]:

(a) thermoplastics (thermoplastic polymers)—under the influence of higher temperature they become plastic and after cooling down they harden again;
(b) duroplastics (thermo-set or chemo-set polymers)—after being exposed to temperature or chemical substance, they become hard, their formation is irreversible;
(c) elastomers—they deform to a large extent under low stress, it is possible to return to their original shape.

One of the most commonly used sealing materials is PTFE (polytetrafluoroethylene) [5,10,11,32,50,51,54,104] or PTFE with added bronze [13,51,54,104]. PTFE with other materials, e.g., carbon, is also mentioned in [104]. The PTFE has high chemical resistance and a very favourable friction coefficient, which translates into minimizing the risk resulting from the stick-slip phenomenon [11].

In [54], pure PTFE was used for piston guide rings, while PTFE with bronze was used for backup rings for pistons and guide ring for the piston rod. In [104], the results showed that pure PTFE, bronze-filled PTFE, and carbon-filled PTFE allow for lower breakaway friction. In [32], the application of PTFE to piston rod guides was described.

In [51], the effect of the operation of seals of different profiles made of PTFE with bronze and PU (PUR, polyurethane) for high operating speeds, such as those found in mining, was examined. The results showed that the seal’s suitability for given operating conditions depends on the seal profile.

The regular PU mentioned in [51] is also a frequently used material for sealing systems [7,8,11,48,52,54,57,105]. It can also be found in thermoplastic version of TPU [9,10,13,50] or as HPU (resistant to hydrolysis) [13].

In [51], PU is used for sealing elements U of the piston as well as for scrapers and main sealing elements in the piston rod sealing system. In [55,105], PU was used for sealing the piston rod.

Another material used for seals is NBR (nitrile butadiene rubber) [9,10,11,54,58,59] and HNBR (hydrogenated nitrile butadiene rubber) [10].

In [54], NBR is used for the main piston sealing element and Glyd sealing of the piston. The papers [58,59] describe the testing of O-rings made of this material. In [9], NBR was described for use on the piston rod wipers.

In [10,13], the application of FPM/FKM for sealing elements of hydraulic cylinders was described. FKM and FPM are the other names for the same material (fluorocarbon rubber). It is resistant to weather conditions and high temperature. It is also characterized by high chemical resistance.

For rings protecting against extrusion of seals in sealing systems of the piston rod, PE (polyethylene) is used [54]. It is a relatively cheap polymer that can be used even at low temperatures.

Other plastic materials used for seals in power hydraulics are: POM [13,73,74], MVQ, EPDM and PE-UHMW [13].

**Figure 10:**
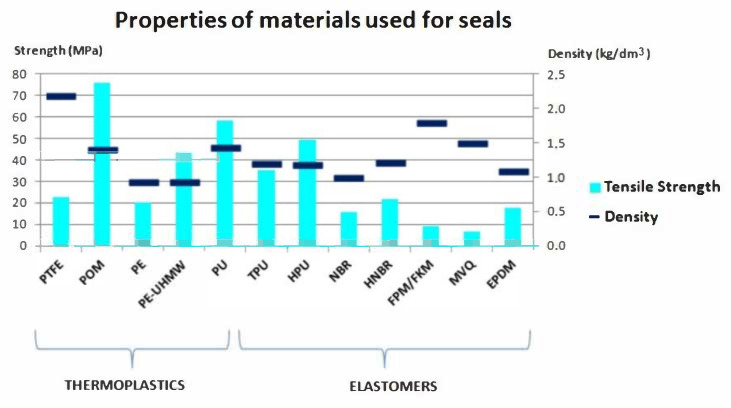
Chart of properties of the materials used for the seals.

Table 9 and Figure 10 show the basic properties of the polymers discussed.

## 5. Conclusions

The article presents a literature review which allowed systematizing the currently available knowledge on materials and methods of surface modifications used in power hydraulics. The issue is quite important, as currently the industry is strongly interested in new materials and technologies for the production of hydraulic system components.

When choosing the appropriate material or surface modification, it is necessary to take into account the working conditions of the component. Hydraulic actuator elements often operate under high loads and are exposed to an aggressive environment. This must be noted as early as at the design stage, because possible failures endangering human health and life and can cause adverse environmental or economic effects.

Currently, there is a significant increase in the use of light alloys or composites in the production of power hydraulics components. This is due to the increasing climate requirements, but also to the greater awareness of manufacturers about environmental protection. The use of modern materials often increases the efficiency of the system and extends the durability of components occurring in it.

Surface parameters of hydraulic actuator parts can be improved in various ways. The area of materials testing and surface modification is almost unlimited, so it is important to look for more environmentally friendly solutions to reduce costs and increase productivity.

## Figures and Tables

**Table 1:** Basic properties of the materials used for cylinders.

Material	Density (kg/dm^3^)	Minimum Yield Strength (MPa)	Minimum Tensile Strength (MPa)	Maximum Carbon Content (%)	Description
St52/S355J2G3/S355JR	7.8	355	490	0.2	low-carbon structural steel
E355				0.22	low-carbon quality steel
S275JR	7.9	275	410	0.21	low-carbon structural steel
S235JR	7.8	235	340	0.2	low-carbon structural steel
BS970070M20	7.8	210	410	0.24	low-carbon structural steel
R35	7.9	235	345	0.16	low-carbon structural steel for pipes
R45	7.9	255	440	0.22	low-carbon structural steel for pipes
IS 1030 GRADE 280-580	7.85	280	580	0.25	non-alloy steel, general purpose
AISI 304	7.9	210	520	0.08	austenitic stainless steel
60-40-18	7.1	276	414	3.4–3.8	spheroidal cast iron
Al 7075-T6	2.8	430 *	510	–	Al-Zn alloy
POM	1.41	67–69	67–85	–	polyoxymethylene
PA	1.13	40	67	–	polyamide
PP	0.92	30	32	–	polypropylene

* For aluminium alloy, instead of the yield strength R_e_, the conventional yield strength R_p0.2_ is given.

**Table 2:** Additional properties of the materials used for cylinders.

Material	Average Hardness	Impact Energy (J)	Elongation at Break (%)
St52/S355J2G3/S355JR	180 HB	27 (−20 °C)	22
E355			
S275JR	160 HB	27 (20 °C)	22
S235JR	140 HB	27 (20 °C)	26
BS970070M20	140 HB	24 (10 °C)	21
R35	112 HB	27 (0 °C)	24
R45	142 HB	22 (20 °C)	22
IS 1030 GRADE 280-580	220 HB	22 (20 °C)	18
AISI 304	215 HB	60 (−196 °C)	45
60-40-18	160 HB	12 (−20 °C)	18
Al 7075-T6	150 HB	17 (23 °C)	10
POM	81 (Shore D)	-	30
PA	76–82 (Shore D)	-	20–200
PP	70–83 (Shore D)	-	150–600

**Table 3:** Basic properties of the materials used for pistons.

Material	Density (kg/dm^3^)	Minimum Yield Strength (MPa)	Minimum Tensile Strength (MPa)	Maximum Carbon Content (%)	Description
65-45-12	7.1	310	448	3.50–3.90	spheroidal cast iron
C35	7.8	270	520	0.32–0.39	medium-carbon structural steel
C45	7.8	305	580	0.42–0.5	medium-carbon structural steel
S275JR	7.9	275	410	0.21	low-carbon structural steel
S355JR	7.8	355	490	0.2	low-carbon structural steel
BS970070M20	7.8	210	410	0.24	low-carbon structural steel
Al 7075-T6	2.8	430 *	510	–	Al-Zn alloy
POM	1.41	67–69	67–85	–	polyoxymethylene

* For aluminium alloy, instead of the yield strength R_e_, the conventional yield strength R_p0.2_ is given.

**Table 4:** Additional properties of the materials used for pistons.

Material	Average Hardness	Impact Energy (J)	Elongation at Break (%)
65-45-12	131–220 HB	14 (23 °C)	12
C35	160 HB	23 (23 °C)	17
C45	200 HB	25 (23 °C)	14
S275JR	160 HB	27 (20 °C)	22
S355JR	165 HB	27 (20 °C)	22
BS970070M20	140 HB	24 (10 °C)	21
Al 7075-T6	150 HB	17 (23 °C)	10
POM	81 (Shore D)	–	30

**Table 5:** Basic properties of the materials used for piston rods.

Material	Density (kg/dm^3^)	Minimum Yield Strength (MPa)	Minimum Tensile Strength (MPa)	Maximum Carbon Content (%)	Description
S235JR	7.8	235	340	0.2	low-carbon structural steel
S275JR	7.9	275	410	0.21	low-carbon structural steel
S355J0	7.8	355	490	0.2	low-carbon structural steel
C45	7.8	305	580	0.42–0.5	medium-carbon structural steel
C35	7.8	270	520	0.32–0.39	medium-carbon structural steel
C55	7.8	330	640	0.5–0.6	medium-carbon structural steel
40Cr/40X	7.8	785	810	0.37–0.44	alloy steel
C45E	7.8	305	580	0.42–0.5	medium-carbon structural steel
40HM/ 42CrMo4	7.8	750 *	1000	0.38–0.45	alloy steel
20MnV6	7.8	410	550	0.22	low-alloy structural steel
19MnVS6	7.8	390	600	0.15–0.22	non-alloy special steel
38MnVS6	7.8	520	800	0.34–0.41	alloy steel
30CrNiMo8	7.8	830	980	0.26–0.34	alloy structural steel
BS970070M20	7.8	210	410	0.24	low-carbon structural steel
BS970070M55	7.8	330	640	0.52–0.6	medium-carbon structural steel
17-4PH	7.8	1000	1100	0.07	martensitic stainless steel
AISI 304	7.9	210	520	0.08	austenitic stainless steel
AISI 410	7.8	415	450	0.15	martensitic stainless steel
Al 7075-T6	2.8	430 *	510	–	Al-Zn alloy
POM	1.41	67–69	67–85	–	poly-oxymethylene

* For aluminium alloy and 42CrMo4 steel, instead of the yield strength R_e_, the conventional yield strength R_p0.2_ is given.

**Table 6:** Additional properties of the materials used for piston rods.

Material	Average Hardness	Impact Energy (J)	Elongation at Break (%)
S235JR	140 HB	27 (20 °C)	26
S275JR	160 HB	27 (20 °C)	22
S355J0	165 HB	27 (0 °C)	18
C45	200 HB	25 (23 °C)	14
C35	160 HB	23 (23 °C)	17
C55	225 HB	25 (23 °C)	15
40Cr/40X	200 HB	47 (23 °C)	9
C45E	207 HB	25 (23 °C)	16
40HM/ 42CrMo4	218 HB	30 (23 °C)	10
20MnV6	220 HB	27 (−20 °C)	19
19MnVS6	255 HB	24 (23 °C)	16
38MnVS6	275 HB	20 (20 °C)	12
30CrNiMo8	250 HB	30 (23 °C)	13
BS970070M20	140 HB	24 (10 °C)	21
BS970070M55	220 HB	25 (23 °C)	12
17-4PH	305 HB	42 (23 °C)	16
AISI 304	215 HB	60 (−196 °C)	45
AISI 410	217 HB	30 (23 °C)	20
Al 7075-T6	150 HB	17 (23 °C)	10
POM	81 (Shore D)	–	30

**Table 7:** Basic properties of the discussed materials used for bottoms and glands.

Material	Density (kg/dm^3^)	Minimum Yield Strength (MPa)	Minimum Tensile Strength (MPa)	Maximum Carbon Content (%)	Description
S355/S355JR	7.8	355	490	0.2	low-carbon structural steel
S275JR	7.9	275	410	0.21	low-carbon structural steel
Al 7075-T6	2.8	430 *	510	–	Al-Zn alloy
IS 1030 GRADE 280-580	7.85	280	580	0.25	non-alloy steel
BS970070M20	7.8	210	410	0.24	low-carbon structural steel
POM	1.41	67–69	67–85	–	poly-oxymethylene
C45	7.8	305	580	0.42–0.5	medium-carbon structural steel
42CrMo4	7.8	750 *	1000	0.38–0.45	alloy steel
AISI304	7.9	210	520	0.08	austenitic stainless steel
G25	7.2	165	250	3.2–3.5	grey cast iron

* For aluminium alloy and 42CrMo4 steel, instead of the yield strength R_e_, the conventional yield strength R_p0.2_ is given.

**Table 8:** Additional properties of the materials used for bottoms and glands.

Material	Average Hardness	Impact Energy (J)	Elongation at Break (%)
S355/S355JR	165 HB	27 (20 °C)	22
S275JR	160 HB	27 (20 °C)	22
Al 7075-T6	150 HB	17 (23 °C)	10
IS 1030 GRADE 280-580	220 HB	22 (20 °C)	18
BS970070M20	140 HB	24 (10 °C)	21
POM	81 (Shore D)	–	30
C45	200 HB	25 (23 °C)	14
42CrMo4	218 HB	30 (23 °C)	10
AISI 304	215 HB	60 (−196 °C)	45
G25	215 HB	12 (−20 °C)	0.5

**Table 9:** Basic properties of the materials used for sealing purposes.

Material	Density (kg/dm^3^)	Minimum Tensile Strength (MPa)	Minimum Service Temperature (°C)	Maximum Service Temperature (°C)	Description
**THERMOPLASTICS**					
PTFE	2.2	17–28	−200	250	polytetrafluoroethylene
POM	1.41	67–85	−50	90	polyoxymethylene
PE *	0.91–0.94/ 0.95–0.98	7–17/ 20–37	−50/−50	75/80	polyethylene
PE-UHMW	0.94	38.6–48.3	−150	90	polyethylene (ultra high molecular weight)
**ELASTOMERS**					
PU	1.45	20.7–96.0	−60	90	polyurethane
TPU	1.2	30–40	−40	80	thermoplastic polyurethane
HPU	1.19	49.2	−30	110	hydrolysis resistant polyurethane
NBR	1.0	6.89–24.1	−30	120	nitrile butadiene rubber
HNBR	1.23	21.7	−30	150	hydrogenated nitrile butadiene rubber
FPM/FKM	1.8	9	−35	220	fluorocarbon rubber
MVQ	1.5	6.4	−60	200	methylvinyl silicone rubber
EPDM	1.1	17.4	−45	125	ethylene-propylene-diene monomer

* Depending on the structure, a distinction is made between low-density PE (first value) and high-density PE (second value).

## Data Availability

Data sharing is not applicable to this article.

## References

[1] Bohman E. Understanding Buckling Strength of Hydraulic Cylinders. The Hydraulics & Pneumatics Article 2017. http://www.hydraulicspneumatics.com/technologies/cylinders-actuators/article/21887243/understanding-buckling-strength-of-hydraulic-cylinders.

[2] Nicoletto G., Marin T. (2011). Failure of a heavy-duty hydraulic cylinder and its fatigue re-design. Eng. Fail. Anal..

[3] Uzny S., Kutrowski Ł. (2019). Strength analysis of a telescopic hydraulic cylinder elastically mounted on both ends. J. Appl. Math. Comput. Mech..

[4] Osiecki A. (1998). Hydrostatyczny Napęd Maszyn.

[5] Bauer W. (2011). Hydropneumatic Suspension Systems.

[6] Luo P., Hu J., Tan S. (2018). Design and Realization of Hydraulic Cylinder. Reg. Water Conserv..

[7] Huang Y., Salant R.F. (2015). Numerical analysis of a hydraulic rod seal: Flooded vs. starved conditions. Tribol. Int..

[8] (1995). Sealing system for piston rods. Seal. Technol..

[9] Peppiatt N., Seals H. (2003). The influence of the rod wiper on the leakage from a hydraulic cylinder gland. Seal. Technol..

[10] McBride T. Seals for Hydraulic Cylinders. The Hydraulics & Pneumatics Article 2019. https://www.hydraulicspneumatics.com/technologies/seals/article/21118898/seals-for-hydraulic-cylinders.

[11] Barth S. Sealing the Deal in Hydraulic Cylinders. P.I. Process Instrumentation Article 2018. https://www.piprocessinstrumentation.com/bearings-seals/article/15564050/sealing-the-deal-in-hydraulic-cylinders.

[12] Uzny S., Kutrowski Ł. (2018). Obciążalność rozsuniętego teleskopowego siłownika hydraulicznego przy uwzględnieniu wyboczenia oraz wytężenia materiału. Modelowanie Inżynierskie.

[13] Kowalski K., Złoto T. (2014). Exploitation and Repair of Hydraulic Cylinders Used in Mobile Machinery. Teka Comm. Mot. Energetics Agric..

[14] Chalamoński M. (2004). Równomierność ruchu tłoka siłownika hydraulicznego. Diagnostyka.

[15] Skowrońska J., Zaczyński J., Kosucki A., Stawiński Ł., Stryczek J., Warzyńska U. (2021). Modern Materials and Surface Modification Methods Used in the Manufacture of Hydraulic Actuators. Advances in Hydraulic and Pneumatic Drives and Control 2020.

[16] Ashby M.F. (2011). Materials Selection in Mechanical Design.

[17] Kijewska A., Bluszcz A. (2017). Analiza poziomów śladu węglowego dla świata i krajów UE. Syst. Wspomagania W Inżynierii Prod..

[18] Harvey F. New Technology Could Slash Carbon Emissions from Aluminium Production. The Guardian Article 2018. http://www.theguardian.com/environment/2018/may/10/new-technology-slash-aluminium-production-carbon-emissions.

[19] Solazzi L. (2021). Stress variability in multilayer composite hydraulic cylinder. Compos. Struct..

[20] Solazzi L. (2020). Design and experimental tests on hydraulic actuator made of composite material. Compos. Struct..

[21] Solazzi L., Buffoli A. (2019). Telescopic Hydraulic Cylinder Made of Composite Material. Appl. Compos. Mater..

[22] Formicola R., Solazzi L., Buffoli A. (2020). The Multi-Parametric Weight Optimization of a Hydraulic Actuator. Actuators.

[23] Madej M., Ozimina D., Pająk M. (2015). Właściwości powłok węglowych uzyskiwanych w procesach fizycznego osadzania z fazy gazowej. Mechanik.

[24] Wang H., Ma G., Xu B., Yong Q., He P. (2017). Design and application of friction pair surface modification coating for remanufacturing. Friction.

[25] Tonelli L., Martini C., Ceschini L. (2017). Improvement of wear resistance of components for hydraulic actuators: Dry sliding tests for coating selection and bench tests for final assessment. Tribol. Int..

[26] Jakubczak H., Rojek J. (2005). Zmęczeniowe pękanie siłowników hydraulicznych. Diagnostyka.

[27] Bednarek T., Sosnowski W. (2010). Practical fatigue analysis of hydraulic cylinders—Part II, damage mechanics approach. Int. J. Fatigue.

[28] Menchen P. (2005). Analiza nierównomierności ruchu tłoka siłownika hydraulicznego. Diagnostyka.

[29] Tomczyk J., Kosucki A. (2007). Hydrostatic drive of the ferry approach bank. Transp. Probl..

[30] Sochacki W., Bold M. (2017). Damped Vibrations of Hydraulic Cylinder with a Spring-damper System in Supports. Procedia Eng..

[31] Zh Aizhambaeva S., Maximova A.V. (2018). Development of control system of coating of rod hydraulic cylinders. IOP Conf. Ser. Mater. Sci. Eng..

[32] Stryczek S. (2013). Napęd Hydrostatyczy.

[33] Danzer E. Guidelines to Avoid Those Hydraulic-Cylinder Headaches. The Hydraulics & Pneumatics Article 2018. https://www.hydraulicspneumatics.com/technologies/cylinders-actuators/article/21887578/guidelines-to-avoid-those-hydrauliccylinder-headaches.

[34] Denisov L.V., Boitsov A.G., Siluyanova M.V. (2018). Surface Hardening in Hydraulic Cylinders for Airplane Engines. Russ. Eng. Res..

[35] Marczewska I., Bednarek T., Marczewski A., Sosnowski W., Jakubczak H., Rojek J. (2006). Practical fatigue analysis of hydraulic cylinders and some design recommendations. Int. J. Fatigue.

[36] Holyakevych A., Orlov L., Pokhmurs’ka H., Andreykiv O., Hrytsai I., Kindratsky B., Kuzio I., Kushnir R., Pavlishche V., Palash V., Panasyuk V., Pokhmursky V., Stotsko Z. (2013). Restoration of the rods of hydraulic cylinders of mining equipment (in Ukrainian). Abstract of the 11th International Symposium of Ukrainian Mechanical Engineers in Lviv.

[37] Tian J., Wang W., Yan W., Jiang Z., Shan Y., Yang K. (2016). Cracking due to Cu and Ni segregation in a 17-4 PH stainless steel piston rod. Eng. Fail. Anal..

[38] Israelson P. Better Steels Make Better Cylinders. The Hydraulics & Pneumatics Article 2016. https://www.hydraulicspneumatics.com/technologies/cylinders-actuators/article/21885260/better-steels-make-better-cylinders.

[39] Moreira D.C., Furtado H.C., Buarque J.S., Cardoso B.R., Merlin B., Moreira D.D.C. (2019). Failure analysis of AISI 410 stainless-steel piston rod in spillway floodgate. Eng. Fail. Anal..

[40] Rütti T.F., Wentzel E.J., Jones D.R.H. (2001). Investigation of failed actuator piston rods. Failure Analysis Case Studies II.

[41] Tian J., Wang W., Yan W., Jiang Z., Shan Y., Yang K. (2017). Microstructure characteristics of segregation zone in 17-4PH stainless steel piston rod. J. Iron Steel Res. Int..

[42] Tavares S.M.O., Viriato N., Vaz M., de Castro P.M.S.T. (2016). Failure analysis of the rod of a hydraulic cylinder. Procedia Struct. Integr..

[43] Knez M., Glodež S., Kramberger J. (2009). Fatigue assessment of piston rod threaded end. Eng. Fail. Anal..

[44] Nefed’ev S.P., Dema R.R., Kharchenko M.V., Pelymskaya I.S., Romanenko D.N., Zhuravlev G.M. (2017). Experience in Restoring Hydraulic Cylinder Rods by Plasma Powder Surfacing. Chem. Pet. Eng..

[45] Boye T., Adeyemi O., Emagbetere E. (2017). Design and Finite Element Analysis of Double—Acting, Double—Ends Hydraulic Cylinder for Industrial Automation Application. Am. J. Eng. Res..

[46] Tao C., Xi N., Yan H., Zhang Y. (1998). Fatigue failure of hold-down bolts for a hyraulic cylinder gland. Eng. Fail. Anal..

[47] Deaconescu T., Daconescu A. (2002). An analysis of the sealing element—Hydraulic cylinder tribosystem. The Annals of University ‘Dunărea De Jos’ of Galaţi, Fascicle VIII: Tribology.

[48] Bae J., Chung K.H. (2017). Accelerated wear testing of polyurethane hydraulic seal. Polym. Test..

[49] Tomasiak E. (2001). Napędy i Sterowania Hydrauliczne i Pneumatyczne.

[50] Papatheodorou T., Hannifin P. (2005). Influence of hard chrome plated rod surface treatments on sealing behavior of hydraulic rod seals. Seal. Technol..

[51] Heipl O., Murrenhoff H. (2015). Friction of hydraulic rod seals at high velocities. Tribol. Int..

[52] Peng C., Ouyang X., Guo S., Zhou Q., Yang H. (2020). Numerical analysis of the traction effect on reciprocating seals in the hydraulic actuator. Tribol. Int..

[53] Thatte A., Salant R. (2009). Transient EHL analysis of an elastomeric hydraulic seal. Tribol. Int..

[54] Pan Q., Zeng Y., Yi-bo L., Jiang X., Huang M. (2021). Experimental investigation of friction behaviors for double-acting hydraulic actuators with different reciprocating seals. Tribol. Int..

[55] Zhao X., He X., Wang L., Chen P. (2020). Research on pressure compensation and friction characteristics of piston rod seals with different degrees of wear. Tribol. Int..

[56] Tran X.B., Hafizah N., Yanada H. (2012). Modeling of dynamic friction behaviors of hydraulic cylinders. Mechatronics.

[57] Kanters A.F.C., Visscher M. (1989). Lubrication of reciprocating seals: Experiments on the influence of surface roughness on friction and leakage. Tribol. Ser..

[58] Wang B., Meng X., Peng X., Chen Y. (2021). Experimental investigations on the effect of rod surface roughness on lubrication characteristics of a hydraulic O-ring seal. Tribol. Int..

[59] Scaraggi M., Angerhausen J., Dorogin L., Murrenhoff H., Persson B. (2018). Influence of anisotropic surface roughness on lubricated rubber friction with application to hydraulic seals. Wear.

[60] Muraki M., Kinbara E., Konishi T. (2003). A laboratory simulation for stick-slip phenomena on the hydraulic cylinder of a construction machine. Tribol. Int..

[61] Rana A.S., Sayles R.S. (2005). An experimental study on the friction behaviour of aircraft hydraulic actuator elastomeric reciprocating seal. Tribol. Interface Eng. Ser..

[62] Dašić P., Manđuka A., Pantić R. (2008). Research of optimal parameters of machining big hydraulic cylinders from the aspect of quality. Annals of the Oradea University—Fascicle of Management and Technological Engineering VII (XVII).

[63] Solazzi L. (2019). Feasibility study of hydraulic cylinder subject to high pressure made of aluminum alloy and composite material. Compos. Struct..

[64] Przybylski W. (2011). Zastosowanie obróbki nagniataniem w technologii siłowników hydraulicznych. Postępy Nauk. I Tech..

[65] Balavignesh V.N., Balasubramaniam B., Kotkunde N. (2017). Numerical investigations of fracture parameters for a cracked hydraulic cylinder barrel and its redesign. Mater. Today Proc. Part A.

[66] Otsima M. (2015). Material Selection Process for Hydraulic Cylinder. https://www.researchgate.net/publication/272506787_Material_Selection_Process_for_Hydraulic_Cylinder.

[67] Pawłowski W., Kępczak N. (2015). Teoretyczne badania właściwości dynamicznych łóż obrabiarki wykonanych z żeliwa i hybrydowego połączenia żeliwa z odlewem mineralnym. Mechanik.

[68] Tubielewicz K., Turczyński K., Szyguła M., Chlebek D., Michalczuk H. (2015). Indywidualny siłownik hydrauliczny wykonany ze stopów lekkich. Mechanik.

[69] Mantovani S. (2020). Feasibility Analysis of a Double-Acting Composite Cylinder in High-Pressure Loading Conditions for Fluid Power Applications. Appl. Sci..

[70] Liu Y., Zwingmann B., Schlaich M. (2015). Carbon Fiber Reinforced Polymer for Cable Structures—A Review. Polymers.

[71] Lubecki M., Leśny J., Chojnicki B.H., Panfil M., Nyćkowiak J. (2019). Selected design issues in hydraulic cylinder made of composite materials. Badania i Rozwój Młodych Naukowców w Polsce: Nauki Techniczne i Inżynieryjne: Materiały, Polimery, Kompozyty.

[72] Scholz S., Kroll L. (2014). Nanocomposite glide surfaces for FRP hydraulic cylinders—Evaluation and test. Compos. Part B Eng..

[73] Stryczek P., Przystupa F., Banaś M. Research on series of hydraulic cylinders made of plastics. Proceedings of the 2018 Global Fluid Power Society PhD Symposium.

[74] Stryczek J., Banaś M., Krawczyk J., Marciniak L., Stryczek P. (2017). The Fluid Power Elements and Systems Made of Plastics. Procedia Eng..

[75] Harnisch M. (2013). Kunststoffe n fluidtechnischen Antrieben. Oelhydraulik Und Pneum..

[76] Dobrzański L.A. (2006). Podstawy Nauki o Materiałach i Metaloznawstwo.

[77] Kula P. (2000). Inżynieria Warstwy Wierzchniej.

[78] Walczak P. (2014). Analiza modelu matematycznego układu sterowania kierownicą turbiny wodnej małej mocy. Logistyka.

[79] O’Rourke B. (2014). Pressure Ratings and Design Guidelines for Ductile Iron Manifolds. 2014 IFPE Technical Conference: Where All the Solutions Come Together and Connections Are Made.

[80] Krawczyk J., Stryczek J. (2013). Układ hydrauliczny z elementami wykonanymi z tworzyw sztucznych. Górnictwo Odkryw..

[81] Wach P., Michalski J., Tacikowski J., Kowalski S., Betiuk M. (2008). Gazowe azotowanie i jego odmiany w przemysłowych zastosowaniach. Inżynieria Mater..

[82] Dai L.-Y., Lin S.-F., Yang S.-Z., Pan G.-F., Guo N., Dai L.-L. (2011). Cracking cause analysis of 45 steel piston rod. Heat Treat. Met..

[83] Bobzin K., Öte M., Linke T.F., Malik K.M. (2015). Wear and Corrosion Resistance of Fe-Based Coatings Reinforced by TiC Particles for Application in Hydraulic Systems. J. Therm. Spray Technol..

[84] Sevagin S., Mnatsakanyan V.U. (2020). Ensuring the required manufacturing quality of hydraulic-cylinder rods in mining machines. IOP Conference Series: Materials Science and Engineering 2019.

[85] Kawiak M. (2013). Spawanie tłoczyska siłowników hydraulicznych. Przegląd Spaw..

[86] Xi Y., Liu D., Han D. (2008). Improvement of corrosion and wear resistances of AISI 420 martensitic stainless steel using plasma nitriding at low temperature. Surf. Coat. Technol..

[87] Tuominen J., Näkki J., Pajukoski H., Miettinen J., Peltola T., Vuoristo P. (2015). Wear and corrosion resistant laser coatings for hydraulic piston rods. J. Laser Appl..

[88] Deng X., Ju D. (2013). Modeling and Simulation of Quenching and Tempering Process in steels. Phys. Procedia.

[89] Awad M., Hultgren J., Roberts W. Increased Resistance to Buckling of Piston Rods Through Induction Hardening, OVAKO Article 2018. http://www.ovako.com/globalassets/products/hard-chromed/ih_buckling_wp.pdf.

[90] Gayathri N., Karthick N., Shanmuganathan V.K., Adhithyan T.R., Madhan Kumar T., Gopalakrishnan J. (2018). Productivity Improvement and Cost Reduction in Hydraulic Cylinders. Int. J. Eng. Technol..

[91] Veeman D., Jagadeesha T. (2020). Tribological characterization of electrolytic hard chrome & WC-C0 coatings. Mater. Today Proc..

[92] Podgornik B., Massler O., Kafexhiu F., Sedlaček M. (2018). Crack density and tribological performance of hard-chrome coatings. Tribol. Int..

[93] Schneider B.C., Constant S.L., Patierno S.R., Jurjus R.A., Ceryak S.M. (2012). Exposure to particulate hexavalent chromium exacerbates allergic asthma pathology. Toxicol. Appl. Pharm..

[94] Vardelle A.M. LCA F18 Hard chrome and HVOF coating. Proceedings of the International Thermal Spray Conference and Exposition 2008—ASM Thermal Spray Society.

[95] Flitney B. (2007). Alternatives to chrome for hydraulic actuators. Seal. Technol..

[96] Bolelli G., Giovanardi R., Lusvarghi L., Manfredini T. (2006). Corrosion resistance of HVOF-sprayed coatings for hard chrome replacement. Corros. Sci..

[97] Picas J.A., Forn A., Matthäus G. (2006). HVOF coatings as an alternative to hard chrome for pistons and valves. Wear.

[98] Hackett C., Settles G. The High-Velocity Oxy-Fuel (HVOF) thermal spray—Materials processing from a gas dynamics perspective. Proceedings of the Fluid Dynamic Conference.

[99] Hutsaylyuk V., Student M.M., Zadorozhna K., Student O., Veselivska H., Gvosdetskii V., Maruschak P., Pokhmurska H. (2020). Improvement of wear resistance of aluminum alloy by HVOF method. J. Mater. Res. Technol..

[100] Holyakevych A.A., Orlov L.M., Pokhmurs’ka H.V., Student M.M., Chervins’ka N.R., Khyl’ko O.V. (2015). Influence of the Phase Composition of the Layers Deposited on the Rods of Hydraulic Cylinders on Their Local Corrosion. Mater. Sci..

[101] Zhuk Y. (2019). Nanostructured CVD Tungsten Carbide Coating on Aircraft Actuators and Gearbox Shafts Reduces Oil Leakage and Improves Durability. J. Mater. Eng. Perform..

[102] Dalibón E.L., Pecina J.N., Moscatelli M.N., Ramírez Ramos M.A., Trava-Airoldi V.J., Brühl S.P. (2019). Mechanical and Corrosion Behaviour of DLC and TiN Coatings Deposited on Martensitic Stainless Steel. J. Bio-Tribo-Corros..

[103] Ashby M.F., Jones D.R.H. (1996). Materiały Inżynierskie.

[104] Golchin A., Simmons G., Glavatskih S. (2012). Breakaway friction of PTFE materials in lubricated conditions. Tribol. Int..

[105] Huang Y., Salant R. (2016). Simulation of a Hydraulic Rod Seal with a Textured Rod and Starvation. Tribol. Int..

